# A degradation index model for maintenance prediction run-to-failure in production systems

**DOI:** 10.1038/s41598-026-56105-4

**Published:** 2026-08-06

**Authors:** Tiago Zonta, Cristiano André da Costa, Felipe André Zeiser, Gabriel de Oliveira Ramos, Rafael Kunst, Rodrigo da Rosa Righi

**Affiliations:** Software Innovation Laboratory (SOFTWARELAB), Applied Computing Graduate Program, Universidade do Vale do Rio dos Sinos (UNISINOS), São Leopoldo, 93022-750 Brazil

**Keywords:** Predictive maintenance, Degradation indexes, Signal similarity patterns, Deep neural networks, Engineering, Mathematics and computing

## Abstract

Timely asset maintenance remains a critical challenge in Industry 4.0 environments. Predictive Maintenance aims to anticipate failures and estimate Remaining Useful Life (RUL), enabling cost reduction and minimizing production downtime. However, real-world industrial scenarios are often characterized by noisy telemetry data, incomplete information about operating conditions, and weak degradation signals, which limit the effectiveness of conventional data-driven approaches. This paper proposes a methodology for RUL prediction under such challenging conditions, leveraging raw sensor telemetry without requiring detailed knowledge of machine operating characteristics. The approach introduces a novel Degradation Index, combined with a Health Index, to better represent degradation patterns. Additionally, signal preprocessing techniques, including Savitzky–Golay and Kalman filters, are applied to mitigate noise and improve data quality. The methodology integrates statistical analysis, similarity-based pattern extraction, and machine learning techniques, including Convolutional Neural Networks and Long Short-Term Memory models, for feature selection and prediction. Experiments conducted on real-world industrial datasets demonstrate that the proposed approach significantly improves prediction performance, achieving high accuracy and enabling failure anticipation up to five days in advance. The results highlight the importance of feature engineering and signal processing in PdM applications, showing that combining degradation modeling with deep learning yields robust, generalizable RUL predictions, even in noisy, partially observed environments.

## Introduction

Technological revolution and human development have altered society’s perception of time. Time appears to be increasingly short in industry, and convergence between manual industrial processes for automation is necessary. In this context, Industry 4.0 (I4.0), a concept involving the evolution and possibility of integrating different technologies and communications, is crucial for the industry’s future^1^. One of the challenges of I4.0 is related to maintenance and the possibility of preventing factory process downtime. Downtime costs can reach half a million euros per day in lost revenue in companies from Belgium, Germany, and Netherlands^2^.

The availability of sensor, data, and machine usage conditions allows run-to-failure analysis, one of Predictive Maintenance’s (PdM) challenges. In run-to-failure analysis, it is possible to anticipate a failure, avoid corrective maintenance, and reduce the occurrence of unnecessary preventive actions^3–6^. In addition, the volume of data helps generate knowledge about machines, processes, resources, plans, quality control, operational, tactical, and strategic costs, as well as measures and key performance indicators (KPIs), such as overall equipment effectiveness (OEE)^7,8^. Furthermore, while existing literature on predictive maintenance and RUL prediction has focused primarily on scenarios with well-characterized machinery and clean datasets, a significant gap remains in methodologies that can effectively handle real-world industrial telemetry data characterized by high noise levels, missing technical specifications, and weak degradation signals^9^. Therefore, our objective with this study is to assist PdM in estimating and predicting machines’ Remaining Useful Life (RUL) using a methodology that can be adapted to general scenarios in the industry.

This article proposes a methodology for predicting RUL using telemetry in noisy data without information on machines’ operation technical features. We used a dataset with telemetry voltage, rotation, pressure, and vibration measurements every hour (cycle) over a year to evaluate our proposal. Our methodology checks and evaluates the quality of the data and creates for each telemetry cycle a Degradation Index (DI) combined with the Health index (HI) proposed by^6^. We used similarity patterns, linear and logistic regression, Savitzky–Golay, Kalman smoothing filters, and the Piecewise function to improve the RUL estimates. Finally, we use machine learning techniques for feature selection and RUL prediction. The scientific contributions of this article are about our strategy for creating a Degradation Index (DI) to penalize smaller variations in the relation between the reading and the expected RUL to guarantee a better adjustment of the model weights. In addition, we understand and relate the importance of analyzing the signals and quantities each sensor can provide. To make the novelty of this work explicit, our contributions are threefold. (i) We propose a Degradation Index (DI) that is computed exclusively from the temporal position of each reading relative to the dataset-wide mean life, and is therefore independent of any failure threshold, physical model, or run-to-failure reference trajectory. This distinguishes the DI from threshold-based degradation indicators and from similarity-based health indices such as those of Wang et al.^6^ and Liang et al.^10^, which require either a known fault threshold or complete healthy-to-failure trajectories that are unavailable in partially observed telemetry. (ii) We combine the DI with a regression-based Health Index (HI) through Principal Component Analysis, yielding a single fused feature (*HI_fitted*) that the feature-importance analysis consistently ranks above the raw sensor channels. (iii) We integrate these engineered features with signal smoothing and a CNN/LSTM pipeline, and we quantify the marginal effect of each stage, so that the gain attributable to the DI, to the DI+HI combination, and to the smoothing filters can be read directly from the reported results. The methodology is deliberately model-agnostic with respect to machine type, which we further support through a cross-domain validation on battery data.

This paper demonstrates that the direct application of Machine Learning (ML) models to raw industrial telemetry often leads to suboptimal results due to data noise, lack of contextual information, and weak degradation patterns. To address this, we conducted a comprehensive study applying strategies that enabled advanced methods such as Convolutional Neural Networks (CNN) and Long Short-Term Memory (LSTM) networks to improve predictive results^11^. We started by evaluating the data and identifying relevant signals, initially testing traditional ML models, which did not produce the desired results. In response, we developed equations that helped identify degradation patterns by incorporating findings from the literature. We also mitigated the effects of noisy data by applying smoothing filters to improve signal quality. The results are presented chronologically to reflect the evolution of our methodology. Furthermore, the proposed approach can be used in other contexts, provided that careful signal analysis and appropriate preprocessing of telemetry data are performed.

The article is divided as follows. Section Background presents the background and related work. Section Related work describes the materials and methods. In Section Materials and methods, we present the results and discussion. Finally, Section Results and discussion highlights the main conclusions and directions for future work.

## Background

I4.0 is based on connectivity, data volume, new devices, miniaturization, inventory reduction, personalization, and controlled production. The differences between traditional factories and the I4.0 model are divided into three prerogatives: components (self-aware, self-predictive); machines (self-aware, self-predictive, self-compare); productive system (self-configure, self-maintain, self-organize)^12^. In this new era, customization and data availability are essential to generate information, which allows actions by people and machines^13^. Therefore, we will discuss concepts related to determining the RUL to enable reliability in the PdM process. We will discuss how to determine data quality and address the use of Deep Neural Networks (DNN) and its related work.

The sensors in I4.0 environments provide new opportunities for PdM solutions^14^. One of the goals in the PdM process is predictive of the RUL that determines the time that the asset can operate without fail^15^. With it, engineers can determine the time to maintenance and optimize efficiency without possible downtime. Hence, the priority of correctly estimating the RUL for PdM processes as well^16^. The idea is that PdM can generate action scheduling based on the equipment’s performance or the conditions over time, resulting in lower maintenance costs, reduced downtime, improved productivity, and quality manufacturing^17^. Advances in ML and the Internet of Things (IoT) leverage PdM by enabling production data analysis and identifying patterns to predict problems before they happen^18,17^. The latest generation of PdM techniques can help reduce downtime by 35% - 45%, maintenance cost by 20% - 25%, and can increase production in 20% - 25%^19^

In this context, we highlighted four approaches for PdM: (i) experience-based: that human experts use years of practical experience in maintaining technical systems^18^; (ii) physical model-based: that the mathematical model reflects on the component condition analysis. Requires the precision of the failure measurement using mathematical and statistical methods to limit these signals^20^; (iii) knowledge-based: approaches that reduce the complexity of a physical model. They are often used as a hybrid strategy, for example, expert systems or with fuzzy logic^21^; (iv) data-driven models: are most commonly found in the current evolution of PdM solutions, based on statistics, pattern recognition, and Artificial Intelligence (AI)^22^.

One of the main requirements for effective PdM is sufficient data from all manufacturing processes^23^. Raw historical data usually has errors and noise, affecting final decision-making. For example, sensor telemetry may not be sufficient to cover critical perspectives of system phenomena or contain redundant information^24^. Among the existing strategies, data based on similarities allows the identification of a degradation pattern in telemetry cycles. Another necessity is understanding how to apply digital, mathematical, and statistical filters for data smoothing^25–27^.

The increase in research related to PdM reflects the industry’s new needs. Challenges imposed by the volumes of data created essential discussions, especially during the period starting in 2018. Approaches linked to ML, DNN models, and Recurrent Neural Networks (RNN) showed promising results in applications with considerable information volumes^28,29^.

## Related work

In this section, we highlight four points explored in the literature: (i) reviews that can bring a broad view of data-driven strategies application for PdM; (ii) examples of datasets commonly used; (iii) application and comparison with ML to predict the RUL; (iv) ways to identify indexes of data degradation using similarity.

We conducted studies to identify PdM challenges in I4.0 to meet the first point. As a result, we can highlight two revisions: one research related to the use of data-driven methods that present a PdM scheme and discuss perspectives of the ML and DNN with five indicators (signal type, application scenario, target, accuracy, and data source)^30^. Another review that discusses ML methods applied to PdM brings contributions to approaches used to different equipment and draws attention to some data collection process strategies for modeling PdM^31^.

Regarding the second point, some datasets are found in challenges and competitions, for example, The Prognostics and Health Management Society (PHM Society). One solution to predict the RUL in the 2008 competition brings an outline for Health Indicator (HI) identification and calculation and a detailed description of the results. With HI, we can calculate degradation characteristics to predict the RUL. The method uses a fusion of telemetry data to use linear regression to predict degradation^6^.

Several articles use the Commercial Modular Aero-Propulsion System Simulation (C-MAPSS) tools that NASA’s Prognostics Center of Excellence provided. Among them, we highlight the application and creation of an HI, reporting a method for forecasting RUL based on similarity^10^. Another paper proposes a similarity-based Particle Filter (PF) and presents results using DNN^25^. Both works bring contributions and have approaches using similarity-based for the RUL prediction. When finding a dataset, it is crucial to assess the quality of the information. We found a method focused on PHM using a visual assessment-based data partitioning method for data quality evaluation and predictive improvement^24^.

Regarding the third point, RNN specifically used for LSTM, we studied the application and comparisons made by^27,32–34^. As already pointed out in previous studies^35^, using LSTM networks is generally related to your long-term memory and not only to recent machine usage characteristics^27^. Another contribution is the use of ensemble methods to improve PdM performance. For example, the article by^36^ presents a method to predict the RUL using the Kalman Filter and Neural Network Ensemble. According to the authors, the Kalman filter can capture statistical uncertainties, which help predict the RUL. A recent research brings an approach highlighting degradation index strategies, smoothing filters, and DNNs and details a new approach called the dual attention and LSTM lightweight model (DA-LSTM)^37^.

Advanced fault prognostics are crucial for improving maintenance planning and avoiding costly shutdowns. The referenced study^11^ highlights a data-driven approach for predicting mechanical failures and estimating RUL using neural networks. Our work shares this focus, aiming to enhance signal quality to improve this process. We present our best results with CNN and LSTM, offering a strategy that can be used in industrial maintenance processes.

Lourari et al.^38^ combine CEEMDAN with sequential backward selection for bearing fault diagnosis under variable operating conditions, while Lourari et al.^39^ employ the Hilbert–Huang transform together with singular value decomposition and supervised learning for bearing and gear faults. Lourari et al.^40^ integrate variational mode decomposition and particle swarm optimisation for multisensory fault diagnosis, and Lourari et al.^41^ derive dedicated health indicators for bearing failures under variable loads, an objective conceptually aligned with our HI/DI construction although applied to vibration signatures rather than to multivariate telemetry without operating-condition labels. For RUL estimation specifically, Lourari et al.^42^ propose a wavelet-packet and ANFIS framework for bearing prognostics. More recently, data-efficient deep learning has gained traction: Saleem et al.^43^ introduce a few-shot learning framework with an attention-augmented backbone that reaches high accuracy under limited labelled data, and Umar et al.^44^ report a deep-learning approach for failure prognosis in an engineering-failure-analysis setting. These works confirm that decomposition, smoothing, and learned indicators remain central to current prognostics research.

Finally, we present the four points we explored in the literature and highlighted in Table 1 the comparative with our contributions. The table brings the differentials used: indexes HI, DI, and smoothing filters to assist in the RUL prediction process. In the following sections, we detail our methodology to present the results of these strategies.

**Table 1:** Comparison of our differentials with the approaches of the related work.

	Dataset	Degradation index(DI)	Health index(HI)	Piece-wise	Filters	Models	Similarity based	Time series	Results
Our proposal	Azure AI guide for PdM solutions	Yes	Yes	Yes	Savitzky –Golay and Kalman Filter	RRF and DNN (CNN, LSTM)	Yes	Yes	Regression coefficient ($R^2$) and accuracy above 90%. We compare RMSE, and we allow failure prediction with considerable anticipation of five days or one hundred and twenty telemetry cycles.
Wang et al.^6^	PHM08 Challenge	No	Yes	No	No	Regression Models and Moving Average Method	Yes	No	The results of the article are guided by competition. The score of the result sent was among the best.
Zheng et al.^27^	C-MAPSS, PHM08 Challenge and Milling	No	No	Yes	No	Multi-Layer Perceptron (MLP), Support Vector Regression (SVR), Relevance Vector Regression (RVR), CNN and LSTM	No	Yes	The results are based on the comparing of the RMSE. Two tables are presented comparing the models used for each dataset.
Dong et al.^33^	C-MAPSS Jet Engine Failure	No	No	No	No	LSTM	Yes	Yes	Results based on RMSE and presentation of RUL graphs.
Bruneo and De Vita^32^	C-MAPSS	No	No	No	No	LSTM	No	Yes	Was made comparisons with literature results and presented the RMSE by reducing it by about 35% and 54% approaches, with the RMSE score equal to 11.42 and a percentage error equal to 0.12.
Liang et al.^10^	C-MAPSS	Yes	No	No	No	Linear Regression, Spearman-coefficient-based model	Yes	-	Results based on MAE and presentation of RUL graphs comparing with two other methods with considerable improvement.
Cai et al.^25^	C-MAPSS	No	No	No	Particle Filter	Rao-Blackwellized Particle Filter (RBPF)	Yes	No	The comparison is with the Benchmarks, and using the RMSE and score criteria, claim that improved prediction compared with other existing RUL prediction methods.
Shi et al.^37^	C-MAPSS	No	Yes	Yes	Exponential Filter	DA-LSTM	Yes	Yes	A detailed proposal for a novel DA-LSTM lightweight model based on exponential smoothing and concerns a can achieve high prediction accuracy with fewer parameters.

Despite the numerous approaches found in the literature, most state-of-the-art models do not effectively handle noisy, non-stationary data from real-world industrial telemetry. Furthermore, while some combine neural networks with smoothing or regression, our method differs by integrating similarity-based degradation indexes with CNN and LSTM models. The combination of DI and HI, when compared with existing literature, enhances the learning capacity under high-noise scenarios.

## Materials and methods

**Fig. 1:**
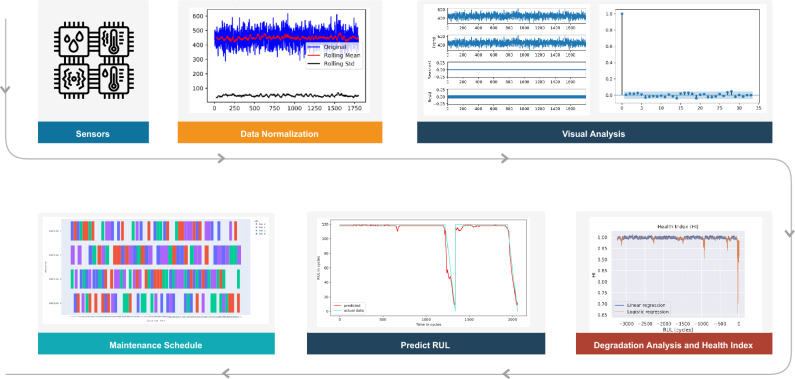
PdM process model applying our methodology. The novel aspects include the creation of a Degradation Index (DI), combination with Health Index (HI), application of smoothing filters, and daily re-evaluation for continuous RUL updates.

With our studies, we delimited what we need to present a model that, through the input of sensor telemetry data, machine information, operation, and workload information, we could predict the moment of failure that allows decision-making. As a result, an overview of the methodology employed in this work is presented in Fig. 1. The methodology is divided into six stages: (i) preprocessing, data evaluation, and testing; (ii) estimate RUL based on available data separation in training, testing, and validation; (iii) visual and statistical analysis of results and application of ML models for PdM, evaluation and creation indexes of degradation; (iv) studies, training, and evaluation of hyperparameters; (vi) we employed daily telemetry reading with new twenty-four cycles to recalculate them and double-check the machine’s situation estimating the new RUL and re-validated the PdM. Finally, we emphasize that our methodology is focused on scenarios with data already collected. Therefore, our objective is to propose a method that can be applied in other cases with few adaptations.

Furthermore, what differentiates our method from existing approaches is the systematic combination of degradation modeling and signal smoothing integrated into a deep learning pipeline. Unlike generic CNN/LSTM applications, our method begins by extracting physically interpretable features that enhance predictive performance in noisy environments.

### Dataset

The initial step in our research involved selecting an appropriate dataset. Previous studies have explored various datasets for PdM applications. However, we focused on identifying a dataset that closely aligns with the industrial contexts where we plan to conduct our initial experiments. To achieve a generalizable approach, we established specific criteria for dataset selection. These criteria included (i) data from diverse machines, (ii) a range of operational durations, (iii) different machine models, and (iv) multiple instances of machine failures during the data collection period. Accordingly, we selected a public dataset hosted on the Microsoft Azure AI Platform that meets these requirements^45^.

The dataset comprises data from 100 machines, including their age and four distinct model identifications. Each machine records hourly telemetry data, encompassing metrics like voltage, rotation, pressure, and vibration from sensors. Notably, the dataset lacks information on operational conditions such as machine setup, production capacity, speed, exertion levels, and environmental factors, which are crucial for understanding sensor correlations. For instance, a simultaneous voltage, rotation, and vibration increase could indicate changes in production capacity or operating regime. The dataset also includes hourly error logs, presented in a binary format without detailed identification, and maintenance records specifying the model of the component serviced, which may or may not have failed. During maintenance, the dataset identifies the failed component and whether it necessitated replacement.

The dataset features the following variables: DateTime, machineID, machineAge, machineModel, voltage, rotation, pressure, vibration, error, comp, and failureCOMP.

To further validate our methodology’s generalizability, we employed an additional dataset focused on battery Remaining Useful Life prediction. This dataset comprises publicly available battery data from Hawai‘i Natural Energy Institute (HNEI), specifically the time series data. Unlike the industrial machinery dataset, this battery dataset presents different challenges: predicting RUL using only voltage (V), current (A), and time (S) measurements, without direct capacity measurements.

We stress that the absence of operating-condition descriptors in the industrial dataset (load, production speed, exertion, and environment) is not merely an inconvenience but a deliberate test condition: it is representative of the large installed base of factories that already record telemetry but do not annotate operating regimes. The practical consequence is that the same raw reading can correspond to different health states under different, unobserved regimes, which inflates aleatoric uncertainty and weakens the correlation between any single sensor and the true RUL. Our engineered indices and smoothing are designed precisely to recover a usable degradation signal under this constraint; nevertheless, as discussed in Section Limitations, this missing context imposes an upper bound on attainable reliability that no amount of feature engineering can fully remove. The battery dataset, by contrast, is acquired under more controlled conditions, and we use it as a complementary, cross-domain check rather than as an exhaustive second benchmark.

### Data pre-processing

The selected dataset is prepared for fail classification. In this way, our first task is to pre-process the data, adapting the dataset to estimate the RUL. The RUL identification is usually modeled as a time-series regression problem due to its characteristics and the sensor’s periodic reading. Therefore, it is crucial to model time-sensitive data correctly. In this case, learning is determined by using time with delay, for example, predicting the current day’s RUL but considering a 10-day data delay. We used a sliding or rolling characteristic, where set size is maintained for predicting *T+1*, and the learning is done always using the closest data. A predefined window size is necessary to input the layer in DNN models.

Another pre-process decision is how to normalize the data and how we will estimate the RUL. It is essential to evaluate mathematical and statistical methods to adjust characteristics used in ML models. There are several ways to normalize sensor data. We aim to scale the raw sensor data within the range [0,1] by using, for example, z-score or min-max normalization^27^. Finally, we have three main ways to estimate the RUL^46^: (i) survival models, in which there is only data on failure time; (ii) degradation model, in which there is information on the fault threshold, data on the moment of machine integrity, and the identification of fault status; (iii) similarity model, in which the information is complete, from the state of integrity to the failure. We analyzed all three scenarios. However, because the dataset does not provide information about the operating conditions, estimating the RUL using the degradation or similarity models was impossible. Consequently, our work was limited to estimating the RUL using the survival model, where we predicted the RUL by cycles to fail. This information is available in the dataset because it identifies the failed component at a given time.

Over a year, the dataset was collected with machine sensor readings (telemetry cycles) of twenty-four hours per day. We split the data into training, validation, and testing in three ways: machine, date, and failures. The division by failures presented the best results, as the machines showcased more than one failure in the time interval. In this division format, the test data showed at least one failure per machine, which was essential to assess the learning of the models. Hence, we created our split algorithm to avoid separate information in each failure. The split resulted in 75% for training and 25% for testing. Of the 75% of training, we selected 5% for models validation. The failure-aware split was chosen specifically so that every machine in the test set contains at least one complete failure episode, which prevents the trivial leakage that a purely random per-cycle split would introduce in time-series data and guarantees that the reported metrics reflect prediction on unseen failure events rather than interpolation within a single trajectory.

To adapt telemetry to our objective, we also grouped telemetry in three ways: (i) per hour, (ii) per three hours, and (iii) per day (twenty-four hours). Furthermore, we created feature statistical measurements by daily mean and standard deviation of voltage, rotation, pressure, and vibration telemetry. Finally, we model our data using a one-hundred-cycle sliding window for each RUL forecast.

### Degradation and health indexes

We use visual and static assessment strategies to help understand the models in noisy data situations. Raw historical data usually has errors and noise, affecting final decision-making. For example, telemetry may not be sufficient to cover critical perspectives on system phenomena or contain redundant information^24^. Based on visual validations, we adopted two indexes to assist in the learning process of the models.

As shown in Fig. 3, the initial results of applying the ML models showed situations of early failure detection. The hypothesis was that the models did not identify small signal changes. In the case of the dataset used, the data presented linear characteristics. The DI was created and calibrated so that some points presented the decay situation so that the models could understand the situation that the telemetry indicated.

The DI, Equation (1), is based on a natural exponential function (*e*). The use of a function with exponential characteristics is motivated by the desired behavior when adjusting the regression model. For example, we aim to penalize minor variations in the ratio between reading and expected RUL. In this way, we can guarantee a better adjustment of the model weight.

The DI was developed because the data characteristics did not provide values that could effectively support the learning of the models, primarily the perceived decay in the signal over time. This decay made it difficult for the models to capture meaningful patterns. To address this, we amplified the math signal to enhance the model’s ability to detect discrepancies in the telemetry data. This adjustment was crucial for the models to understand anomalies and situations that demonstrated the machine would be in a degradation process.

1 $$\begin{aligned} DI = e^{1.0 - (\frac{cycle}{mean(RUL)})} \end{aligned}$$

In Equation (1), the variable *cycle* represents the current telemetry cycle (time), and *mean(RUL)* is the average Remaining Useful Life for all failures in the dataset. The exponential decay penalizes small variations closer to failure, reinforcing the learning of subtle degradation behavior.

The exponential form is not arbitrary. Writing $r = cycle/\mathrm {mean(RUL)}$ for the normalised age, the index $DI = e^{\,1-r}$ has unit value at the mean life (*r=1*), grows for younger components (*r<1*), and decays for components past the mean life (*r>1*). Its derivative $\textrm{d}(DI)/\textrm{d}r = -e^{\,1-r}$ is largest in magnitude for small *r* and shrinks as *r* increases, so equal increments of age produce larger changes in the target early in life and progressively smaller changes near failure. Under a squared-error objective this is equivalent to placing greater weight on the gradual early-degradation phase, which counteracts the tendency of the raw models to declare failure prematurely when they encounter a noisy spike (the behaviour observed in Fig. 3a). The DI therefore acts as a monotone, smoothly differentiable re-parameterisation of time-to-failure rather than as a learned quantity, which is what makes it stable in the presence of noise. Conceptually, it differs from classical degradation or similarity indicators in that it requires neither a calibrated failure threshold (as in degradation models) nor a library of complete reference trajectories for distance computation (as in similarity-based models such as Liang et al.^10^ and Cai et al.^25^); it depends only on a single global scalar, $\mathrm {mean(RUL)}$, estimated from the observed failure cycles. This makes the DI applicable to the survival-model setting of this work, where threshold and full-trajectory information are unavailable.

We evaluated the application of an HI strategy, Equation (2), performing a linear and logistic regression based on the work proposed in^6^. For this process, we only use original telemetry data: voltage, rotation, pressure, and vibration with cycles per hour.

2 $$\begin{aligned} HI = \alpha +\beta ^{T}.x+\varepsilon =\alpha +\sum _{i=1}^{N}\beta _{i}x_{i}+\varepsilon \end{aligned}$$

In Equation (2), $x_i$ are the normalized sensor measurements at cycle *t*, *α* and $\beta _i$ are regression coefficients learned from the data, and *\varepsilon* is the residual error. The linear regression provides a baseline degradation trend across sensors, while the logistic regression captures more abrupt decay patterns.

The equation proposed in^6^ has *x* as the dimensional feature vector *N*, *HI* is the health indicator, ($\alpha ,\beta$) is *N + 1* model parameters , and *\varepsilon* is the noise term. The process performs a telemetry fusion and uses logistic regression. A distortion of the normal, exponential degradation pattern is reported. For this reason, linear regression was also chosen in the proposed methodology. Unlike the method used by^6^, the data used in this work have more than one failure, so information on average failure cycles was included, predicting a change in results.

After HI creation, we used RRF feature importance functionality for double-checking the feature selection and data adaptation with the regression. We realized that data related to errors and component maintenance is not as necessary as in the example of classification proposed in^45^. For the regression, what stood out was the daily telemetry, model, age, and, especially, the indexes that we created from telemetry.

Finally, we combined the indexes using the functionality of the sklearn decomposition.PCA library that applies the dimensionality reduction of the vectors using PCA along with the numpy.empty_like method. According to^47^, PCA transforms linearly correlated components of the x-pattern into a set of principal components not linearly correlated, and the number of main components may be less than or equal to the number of original components. In addition, this process created a new feature called HI_fitted.

The role of combining two indices rather than using the HI alone is complementary, and this is reflected in our experiments. The HI summarises the instantaneous multivariate sensor state through regression, but, being a static fusion of the current reading, it inherits the weak and noisy decay of the raw signals, and the models built on it preserved the premature-failure behavior of Fig. 3a. The DI injects the temporal, monotone degradation prior described above, which is what removed the spurious early detections; conversely, the DI alone carries no sensor information and cannot localize which machine state corresponds to a given age.

Our methodology highlights the creation and combination of DI and HI. We still have an online RUL checking strategy for each daily telemetry reading with twenty-four new cycles. Therefore, we can recalculate them and double-check the machine’s situation, estimating the new RUL.

### Smoothing filters

Industrial telemetry is often affected by noise and uncertainty, which can severely degrade model accuracy if not addressed properly. Noisy signals may mask degradation trends or introduce false patterns. Our methodology explicitly tackles this by applying smoothing filters and constructing signal-based degradation indexes, allowing more robust learning even under adverse data conditions.

We also evaluated the impacts on the results of applying smoothing filters. We use smoothing filters for signal treatment and noise removal, so we conduct tests to assess their effects on telemetry^6,25,36,48,]^. Based on the literature, we selected Kalman and Savitzky-Golay filters. According to^48^, Savitzky-Golay is a method based on the moving average value known as convolution. It uses the linear least-squares to fit adjacent points to a low-grade polynomial. According^36^, the Kalman filter its most common application is as a forward pass state estimator. The filter predicts the hidden states for the next step, given the history of estimated states and observing noisy outputs. Predicted states are considered optimal, as the filter aims to minimize uncertainties. Therefore, filters are used to verify whether telemetry smoothing could improve the failure prognosis or change model learning. The idea of its use is related to our perception of noise reduction when we created the degradation indexes.

In addition to the notes found in the literature, the use of filters was an issue raised in an interview with engineers who used filters to remove noise and improve the visualization of threshold situations. We interviewed some companies that observe the signals and use monitoring alerts, and in some situations, the filters improve the results. In our article, filters were another strategy tested precisely to evaluate how much they could contribute. According to specific tests performed, removing some noise and using degradation indexes significantly improved the results. This contributed to our research proposal of evaluating the maximum number of situations in search of presenting strategies that can be used.

### Models

**Fig. 2:**
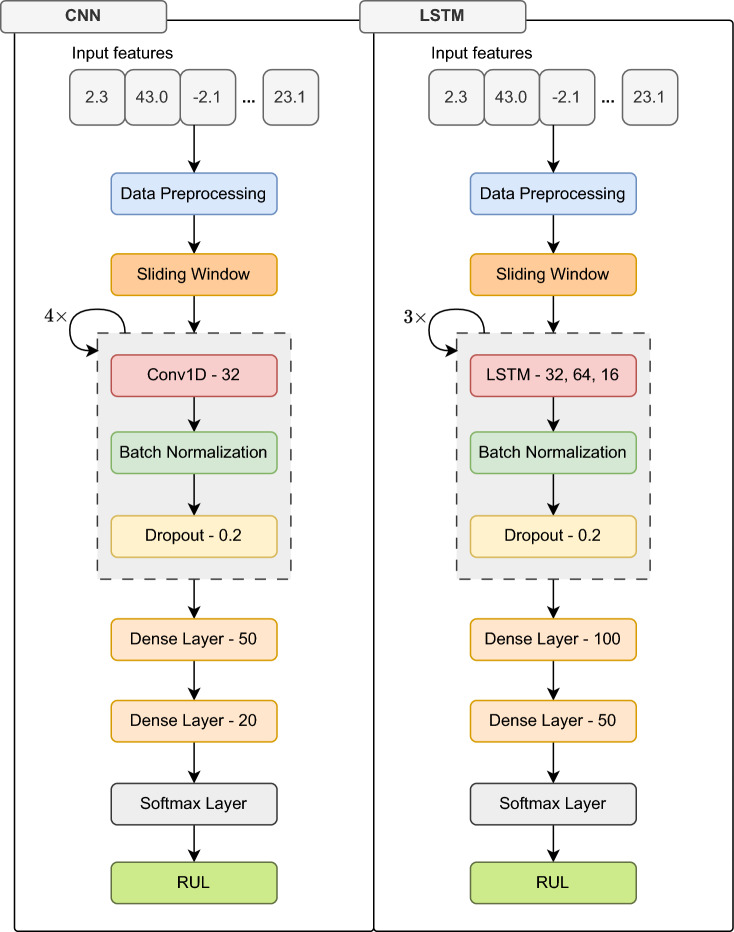
Architecture with the best result applied CNN and LSTM models.

For the prediction of RUL, we evaluated models based on: CNN and LSTM networks. In Fig. 2, we present each evaluated model’s characteristics. We highlight the best results, summarizing our experiments with several tests over six months. In the CNN, we employ four Conv1D Layers = 32, Kernel size = 6, bias and regularizer configuration, and two Dense output layers. Finally, in LSTM, we use three layers = 32-64-16 with the configuration of bias and regularizers, and we also use two Dense layers for output.

For reproducibility, we detail the full configuration here and in the schematic of Fig. 2. The input to both networks is a tensor of shape *100 × F*, where 100 is the sliding-window length (one hundred telemetry cycles) and *F* is the per-cycle feature vector, composed of the daily mean and standard deviation of the four sensors (voltage, rotation, pressure, vibration), the categorical machine model and age, and the engineered *HI_fitted* feature; features are min–max scaled to [0, 1] before windowing. The CNN stacks four one-dimensional convolutional layers with 32 filters each and kernel size 6, each using ReLU activation and $L_2$ kernel and bias regularisation, followed by two fully connected (Deep Feed Forward, DFF) layers whose final unit produces the single continuous RUL output with a linear activation. The LSTM stacks three recurrent layers with 32, 64, and 16 units, respectively, with the same regularisation scheme, followed by two dense output layers. Both networks were trained with the mean squared error (MSE) loss, the RMSProp optimiser, a batch size of 200, and a validation split of 0.05. Hyperparameters (loss, activation, optimiser, and batch size) were selected by grid exploration over the validation split, and the same configuration was kept fixed across the filter and cross-domain comparisons so that performance differences are attributable to the data treatment rather than to retuning.

We select CNNs and LSTMs because of their unique strengths in handling the characteristics of our dataset. CNNs are particularly effective for processing data with spatial hierarchies and patterns, making them well-suited for our industrial data. Their ability to automatically extract features from raw data lets us capture signal patterns that traditional methods might overlook. In the LSTMs case, they are ideal for our time-series analysis of telemetry data. Their architecture allows them to learn long-term dependencies, which is crucial for understanding the progression of degradation over time. By combining CNNs for feature extraction and LSTMs for sequence prediction, our approach maximizes the strengths of both architectures to improve predictive accuracy.

### Evaluation criteria

Our evaluation criteria primarily focused on the coefficient of determination $R^2$ to evaluate the effectiveness of our methodology. $R^2$ measures how well a linear statistical model fits the observed values, in this case, the RUL estimate by our telemetry, providing insights into how much of the variance in the data is explained by the model^17,19^.

We chose $R^2$ due to its effectiveness in evaluating regression models, especially when the goal is to predict continuous variables like RUL. Therefore, we used the r2_score functionality from the sklearn library. According to^49^, the r2_score indicates the fit quality by measuring how well the model predicts samples through the proportion of variance.

Equation (3) inputs the test data (*y*) and the prediction results ($\hat{y}$), with the mean of the actual values $\bar{y}$ defined in Equation (4). In addition to $R^2$, we calculated Accuracy using the Mean Absolute Percentage Error (MAPE) as shown in Equation (6) and evaluated errors using Root Mean Square Error (RMSE), Mean Absolute Error (MAE), and Mean Squared Error (MSE). While $R^2$ measures the overall fit of the model, MAPE captures the percentage accuracy of the predictions, and RMSE, MAE, and MSE provide a comprehensive understanding of the error magnitude and distribution. Together, these metrics offer an important evaluation of model performance, helping to assess not only how well the model fits the data but also the reliability of its predictions in real-world applications. We emphasize that all of these are point-estimate metrics: they characterize the accuracy and error magnitude of a single predicted RUL value but do not express the confidence of that prediction. In safety-critical maintenance, a calibrated predictive interval is often as important as the point estimate.

3 $$\begin{aligned} R^{2}\left( y, \hat{y}\right) = 1 - \frac{\sum _{i=1}^{n}(y_{i}-\hat{y}_{i})^{2}}{\sum _{i=1}^{n}(y_{i}-\bar{y}_{i})^{2}} \end{aligned}$$

4 $$\begin{aligned} \text {where } \bar{y} = \frac{1}{n}\sum _{i=1}^{n}y_{i} \text { e }\sum _{i=1}^{n}(y_{i}-\hat{y}_{i}) = \sum _{i=1}^{n}\epsilon _{i}^{2} \end{aligned}$$

5 $$\begin{aligned} MAPE = \frac{1}{n}\sum _{t=1}^{n}\left| \frac{Actual_t - Forecast_t}{Actual_t}\right| \end{aligned}$$

6 $$\begin{aligned} Accuracy = 100*(1 - MAPE) \end{aligned}$$

## Results and discussion

This section presents the results obtained in test data for models based on CNN and LSTM models. We used different ML methods, analyzed each technique’s characteristics, and indicated which was the most suitable for our data set. In the training, validation, and test, we used a computer with an Intel Core i7-9700K processor, 32 GB of RAM, and an NVIDIA Titan XP with 12 GB of memory.

There were 876,101 telemetry records in which we used time series with a continuous delay window in which the data has a sliding or rolling characteristic, in which the set size is maintained for the prediction of *time(T) + 1*, and learning is always done using the closest data. Therefore, the continuous window with delays of 24, 35, 60, 90, and 100 cycles was chosen, referring to 1, 1.45, 2.5, 3.75, and 4.1 days to predict *T + 1*.

The computational efficiency depends on the hardware used. Initially, testing on a Personal Computer (PC) resulted in a processing time of approximately 20 minutes. This was significantly reduced to around 2 minutes when using a Graphics Processing Unit (GPU), with subtle variations during model tuning and testing hyperparameters.

**Fig. 3:**
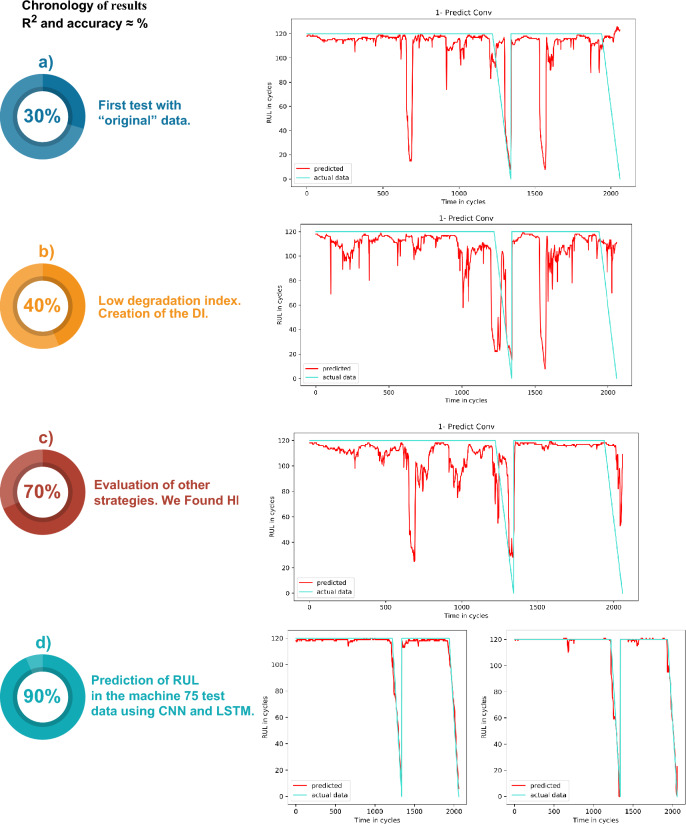
Chronology of results and tests performed. In the icons, we show the accuracy for each test.

To evaluate this issue, we conducted initial experiments using the original dataset without applying any degradation indexes or feature engineering. These tests aimed to assess whether sensor telemetry alone could yield reliable RUL predictions. In effect, the experiments reported in this section constitute a staged ablation of the pipeline: we begin with raw telemetry, then add the engineered indices (DI and HI), and finally add the smoothing filters, keeping the network architecture and hyperparameters fixed throughout so that the change in each metric isolates the effect of the corresponding component. However, the results were poor, with $R^2$ and accuracy values below 30%, and RMSE values ranging from 60 to 90 cycles (hours). Figure 3/a illustrates the prediction for Machine 75, which experienced two failures during the monitored period. The model, implemented using a CNN architecture, exhibited premature failure detection, likely due to noise in the dataset that misled the learning process.

Given these initial findings, we proceeded with the methodology outlined in Fig. 1, starting with data engineering. First, we applied basic normalization techniques and calculated the daily mean and standard deviation for each sensor based on hourly telemetry cycles. We then employed Random Forest Regression (RRF) to evaluate the predictive power of the original features. Once again, performance metrics showed low results. To better understand the data, we conducted visual and statistical analyses, including autocorrelation plots and assessments of stationarity, seasonality, and trends. The results confirmed that the telemetry data were stationary, a property generally favorable for model training. However, the seasonal and trend analyses revealed weak indicators of failure, suggesting that the raw signals lacked strong degradation signatures capable of supporting accurate RUL predictions.

We adjusted the DNN models to test our methodology and tested different hyperparameters for loss, activation, optimizer, and batch size functions. We understand that cases might have different requirements and restrictions. For example, in the case of Loss Function = MSE. The scoring loss function proposed in^27^ was tested, but MSE showed more stable results. The results presented used: Loss Function = mean squared error (MSE), layers NN activation = linear, layers Deep Feed Forward (DFF) activation = relu, optimizer = RMSProp, validation_split = 0.05 e batch size = 200.

When analyzing the data based on our methodology, we realized a failure characteristic in an average of seventy-five days, equivalent to one thousand and eight hundred telemetry cycles. However, the models presented better results considering a Piecewise of one hundred and twenty cycles limit, which gave us a failure forecast five days in advance. We based all tests on this scenario because when based on telemetry analysis and the combination of degradation indexes, we realized that signs of machine degradation start very close to failure.

During the adequacy of the hyperparameters, we noticed considerable improvements. When we applied it to the proposed architecture, combining the two indexes, the results were 70% to 90% of $R^2$, and accuracy and RMSE of 3 to 17 difference cycles as seen in Fig. 3b,c. The chart demonstrates meaningful learning even in visually comparing current data and what was predicted. Furthermore, the prediction process identifies possible failures outside the period, no longer confusing, indicating that the combination of the two indexes significantly reduced noise for RUL prediction.

Figure 3d presents prediction test results for Machine 75 of one hundred and twenty cycles or five days in advance using CNN and LSTM, our best-performing DNN tests. Two failures with different periods demonstrate network adaptation even when estimating the alternating RUL.

**Fig. 4:**
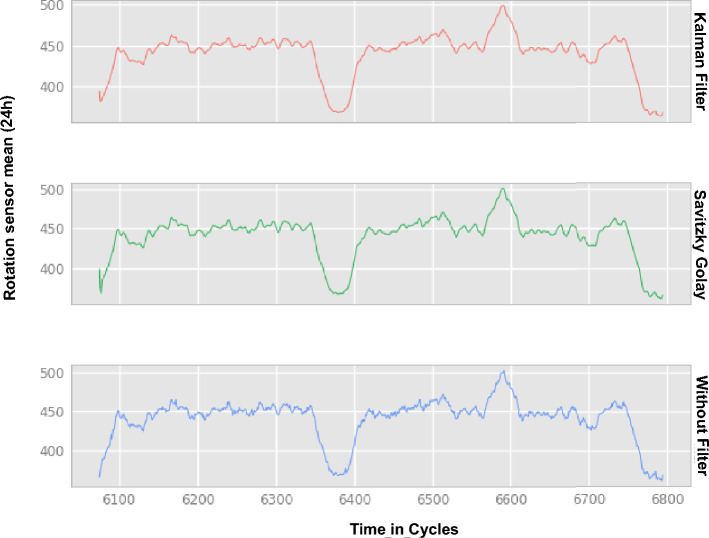
Comparison of signals without and with filters of the daily mean of rotation of the machine 50 test data.

Even with satisfactory results, we observed the possibility of improving our results by applying smoothing filters. Figure 4 shows an example of the daily mean of the machine 50 rotation telemetry. Even though it is difficult to perceive visually, there is improvement in the signals and smoothing at peaks, contributing to the learning of the model. In addition, we remove the sensors’ sampling noise because ML algorithms may associate this noise with important information for RUL prediction. Our goal is to improve failure prediction further or alter model learning.

In Table 2, we present each criterion to visualize the results, testing three situations: without and with the application of smoothing filters in telemetry. This validation is essential because we verify if the telemetry changes with filters, as observed when we created the degradation indexes. For example, in the index, we noticed a considerable decrease in noise, which proved crucial for our results.

**Table 2:** Comparison of model performance under different configurations, highlighting the impact of applying smoothing filters.

Result	Model	RMSE	MSE	MAE	$R^2$	MAPE
Without filter	CNN	9.301	86.503	2.419	0.830	90.85
	LSTM	10.804	116.728	2.861	0.77	90.53
Kalman filter	CNN	9.669	93.484	2.504	0.816	90.64
	LSTM	11.943	142.63	3.308	0.719	90.09
Savitzky-Golay	CNN	8.789	77.253	2.262	0.848	92.22
	LSTM	11.409	130.159	3.159	0.744	91.08

The hyperparameters used for the models were kept constant to compare the results with or without filters. Analyzing all tests, applying the Savitzky-Golay filter is the most consistent result obtained with CNN. Another important point we identified is the behavior of the results for the LSTM network. The results for LSTM (*RMSE=11.409*, *MSE=130.159*, *MAE=3.159*, $R^2=0.744$, *Accuracy=91.08*), compared to the related works, were below expectations^27,32–34,]^.

Compared to LSTMs and other DNNs, CNN’s superior results may be related to CNN’s ability to adapt to noisy data. In addition, CNNs can extract deep information independent of temporal connections^50^. Another factor was the use of DFF to output network layers. We noticed isolated DFF applications did not present satisfactory results (*RMSE=14.19*, *MSE=201.342*, *MAE=11.249*, $R^2=0.604$, *Accuracy=83.81*). Additionally, when used alongside DNNs, their performance improved. The DFFs, in addition to grouping characteristics extracted by the DNNs during data inference, brought nonlinear results and allowed peaks, identifying values close to the minimum and maximum of the RUL^17^.

In the results, applying telemetry filters, we identified a change only in some points. This was because after generating the health indexes, the importance of learning is concentrated on the features created. Nevertheless, the results already present the removal of noise and highlight the characteristics of decay. We can see that the Savitzky-Golay filter allows improvement, and its smoothing is better adapted to our telemetry. On the other hand, the results show that the Kalman Filter could solve situations with more complex noise^36^.

We realized the importance of learning from the created resources when analyzing the results. The results with the degradation indexes already show noise removal and highlight the decay characteristic. However, applying the Savitzky-Golay filter allowed for improvements, and its smoothing fitted the telemetry of the data set used. The results brought a statistical improvement between 2% to 3% in $R^2$ and accuracy due to the smoothing of some peaks.

Our work contributes to understanding that expecting results only from basic data engineering and normalization is an error. We had to apply some essential strategies related to the forecasting process of data improvement through similarity. We presented the chronology in Fig. 3 precisely to demonstrate the situation of obtained results only using sensor telemetry. Our work demonstrated our approach using smoothing filters and feature creation that corroborate the prediction process. The chart indicates that the models were confused by the noise and presented failure at incorrect times. After applying our methodology, we created degradation features and generated the evolution of the results in Fig. 3.

To demonstrate the generalizability of our methodology, we applied our approach to a battery RUL prediction dataset. In the Table 3 we show the results for the battery RUL prediction dataset.

**Table 3:** Cross-domain validation results comparing industrial machinery and battery RUL prediction performance.

Result	Model	RMSE	MSE	MAE	$R^2$	MAPE
Without filter	CNN	12.444	154.797	3.403	0.696	90.41
	LSTM	11.130	123.876	4.128	0.756	88.33
Kalman filter	CNN	9.096	82.735	1.605	0.837	92.49
	LSTM	9.645	93.030	3.724	0.817	89.13
Savitzky-Golay	CNN	3.913	15.310	0.686	0.969	97.50
	LSTM	9.868	97.372	2.792	0.808	90.30

The validation of our methodology on a battery RUL prediction dataset provides strong evidence for its generalizability across different industrial domains. The battery dataset presented unique challenges, requiring RUL prediction using only voltage, current, and time measurements without direct capacity readings. Despite these constraints, our methodology achieved good results, particularly with CNN models (R² = 0.969, Accuracy = 97.5%). The superior performance on the battery dataset compared to the industrial machinery dataset can be attributed to several factors: (1) battery degradation patterns may be more predictable and follow clearer exponential decay patterns, making them more suitable for our degradation index approach; (2) the engineered features from voltage, current, and time measurements may provide cleaner signals with less noise compared to industrial sensor telemetry; (3) battery systems typically have more controlled operating conditions compared to industrial machinery.

Read as an ablation, the chronology of Fig. 3 and Tables 2 and 3 quantify the contribution of each component. Raw telemetry yields $R^2$ and accuracy below 30% with RMSE of 60–90 cycles; adding the DI and HI moves the models into the 70–90% $R^2$ regime with RMSE of 3–17 cycles and, crucially, eliminates the premature-failure artifact; adding the Savitzky–Golay filter contributes a further, smaller improvement of roughly 2–3% in $R^2$ and accuracy (CNN $R^2$ from 0.830 to 0.848). The dominant gain therefore comes from the engineered indices, with smoothing acting as a refinement once the degradation signal has been made explicit.

A legitimate concern is whether the models learn genuine degradation dynamics or merely exploit the engineered signals. Two observations argue for the former. First, the DI and HI are computed solely from quantities available at inference time, the elapsed operating cycle, the min–max-scaled sensor readings, the machine age, and the model identifier, together with a single global constant, $\mathrm {mean(RUL)}$; they are not derived from the per-episode RUL target, so the network is not given a proxy of the answer. Second, the same index construction, applied unchanged to the battery dataset, transfers to a physically distinct system and again improves prediction; an artifact specific to the industrial telemetry would not be expected to generalize in this way.

With respect to benchmarking, our LSTM results (*RMSE=11.409*, $R^2=0.744$) are below the best figures reported on curated benchmarks such as C-MAPSS^27,32–34,]^, whereas our CNN configuration is competitive. We stress that these numbers are not directly comparable: the cited works operate on simulated or run-to-failure data with known operating regimes, while our evaluation deliberately targets noisy field telemetry without operating-condition labels, a harder and less curated setting. Recent signal-decomposition and learned-indicator approaches^38–42^ report strong results but likewise rely on controlled fault injection or condition labels. A like-for-like comparison would require re-implementing these baselines on the present, unlabelled dataset, which we identify as valuable future work.

On computational cost and deployment, the proposed models are intentionally lightweight: the CNN comprises four 32-filter Conv1D layers and the LSTM three layers of 32/64/16 units, which keeps the parameter count small enough to train in roughly two minutes on a single GPU (about twenty minutes on CPU) over the full 876, 101-record series. Inference for a one-hundred-cycle window is effectively instantaneous, so the online step of re-reading twenty-four new cycles per day and recomputing the RUL is trivial to sustain on commodity edge or factory hardware. The main offline cost is the periodic regression, and PCA is used to refresh HI_fitted; both scale linearly with the number of records and do not require specialized accelerators.

Finally, the five-day (one-hundred-and-twenty-cycle) anticipation horizon was observed consistently across the test machines, each of which contains at least one failure episode, including the alternating two-failure case of Machine 75 in Fig. 3d. We present it as an empirically supported horizon for this dataset rather than as a universal guarantee: because the dataset lacks operating-condition labels, we cannot yet stratify the horizon by workload or environment, and validating it across a larger and more heterogeneous fleet under varying conditions is part of the planned work in Section Limitations.

Finally, we aim to encourage PdM adoption in factories with telemetry but still apply corrective or preventive maintenance. We describe our process to validate, create, and combine degradation indexes. In this process, we verify data quality based on visual and statistical methods to present results that can be applied to data that is usually noisy and does not present signs of identification of degradation.

### Limitations

While the results support the value of explicit degradation modeling for noisy telemetry, several limitations should define our next steps. (i) *Missing operating context.* The industrial dataset provides no workload, speed, or environmental labels; this raises aleatoric uncertainty and caps the attainable reliability, and it prevents us from conditioning the model on the regime. (ii) *Validation protocol.* We rely on a single failure-aware 75/25 hold-out with a 5% validation split. A *k*-fold or rolling-origin cross-validation over failure episodes, with confidence intervals per metric, would more rigorously characterize variance and robustness. (iii) *Ablation granularity.* The component contributions are reported sequentially. A full factorial ablation (HI only, DI only, DI+HI, with and without each filter) would quantify interactions more precisely. (iv) *Generalization evidence.* Generalization rests on one industrial dataset plus one cross-domain battery dataset; the battery analysis is a complementary check rather than an exhaustive second benchmark, and broader evaluation across more datasets and machine types is needed. (v) *Baselines.* A like-for-like re-implementation of recent decomposition- and indicator-based methods on this unlabelled dataset is still outstanding. (vi) *Uncertainty.* The reported metrics are point estimates; calibrated predictive intervals (e.g. via Monte Carlo dropout, deep ensembles, or quantile regression) are not yet provided. (vii) *Anticipation horizon.* The five-day horizon is empirically supported on the available test machines but has not been stratified across heterogeneous operating conditions. We regard items (ii), (iii), (v), and (vi) as directly actionable in a follow-up study and items (i), (iv), and (vii) as dependent on access to richer, labeled field data.

## Conclusion and future work

Our proposal presented a methodology for RUL prediction. We started with the hypothesis that a sensor telemetry dataset would be useful without important information about operating conditions and with noise. As a result, we have a 5-day prediction of failure. Expressive results when compared to the literature, as it commonly brings results related to identifying anomalies and predictions based on threshold^35^.

An essential point in our results is the use of heterogeneous data with more than one failure per machine, which led to differences in the calculation of the degradation index. We focus on the idea that relying on sensor data and expecting satisfactory results is an erroneous practice. We have to identify and explain to industries that the mere collection of telemetry data does not support intelligent, assertive predictions.

In summary, our proposal presented a complete cycle for making predictions. We used several strategies and created a Degradation Index that, when combined with other features, yielded satisfactory, applicable results. We also performed comparisons and presented the results of regression and DNN models, in addition to evaluating the improvement of smoothing filter applications using Savitzky–Golay and Kalman filters. Everything we presented can be easily extended to situations in industries with telemetry, but that do not yet use PdM.

In future work, after fulfilling the objective of documenting our results with PdM, we can conduct the studies and propose the integration with Scheduling Problems, thus creating Predictive Maintenance & Schedule (PdMS). With this, we will be able to evaluate a real application in the Industry. Furthermore, we want to reduce downtime, improve communication between the maintenance and production sectors, and enable future integration with the production, storage, and logistics sectors. Another important future direction involves integrating uncertainty quantification into the prediction process. This would allow the model not only to estimate RUL but also to express the confidence or potential variance in its predictions, which is essential in high-stakes industrial environments.

## Data Availability

We used a public dataset hosted on the Microsoft Azure AI Platform (AI 2020) - https://docs.microsoft.com/pt-br/azure/machine-learning/team-data-science-process/predictive-maintenance-playbook#solution-templates-for-predictive-maintenance

## References

[1] Lee, J., Azamfar, M. & Singh, J. A blockchain enabled cyber-physical system architecture for industry 4.0 manufacturing systems. *Manuf. Lett.* **20**, 34–39. 10.1016/j.mfglet.2019.05.003 (2019).

[2] Haarman, M., Mulders, M. & Vassiliadis, C. Predictive Maintenance 4.0: Predict the unpredictable (2017).

[3] Ayvaz, S. & Alpay, K. Predictive maintenance system for production lines in manufacturing: A machine learning approach using iot data in real-time. *Expert Syst. Appl.* **173**, 114598 (2021).

[4] Liu, Z. et al. A novel time series model-based indicator for bearing degradation monitoring. In *Equipment Intelligent Operation and Maintenance* 692–701 (CRC Press, 2025).

[5] Nikolakis, N., Papavasileiou, A., Dimoulas, K., Bourmpouchakis, K. & Makris, S. On a versatile scheduling concept of maintenance activities for increased availability of production resources. *Procedia CIRP* **78**, 172–177. 10.1016/j.procir.2018.03.033 (2018).

[6] Wang, T., Yu, J., Siegel, D. & Lee, J. A similarity-based prognostics approach for remaining useful life estimation of engineered systems. In *2008 International Conference on Prognostics and Health Management, PHM 2008* 1–6. 10.1109/PHM.2008.4711421 (2008).

[7] Ansari, F., Glawar, R. & Nemeth, T. Prima: a prescriptive maintenance model for cyber-physical production systems. *Int. J. Comput. Integr. Manuf.* **32**(4–5), 482–503. 10.1080/0951192X.2019.1571236 (2019).

[8] Saeed, A. et al. Hybrid framework for remaining useful life (rul) prediction of rolling bearing faults. *Array* **2025**, 100498 (2025).

[9] Zhou, J., Yang, J., Xiang, S. & Qin, Y. Remaining useful life prediction methodologies with health indicator dependence for rotating machinery: A comprehensive review. *IEEE Trans. Instrum. Meas.* (2025).

[10] Liang, Z. et al. A degradation degree considered method for remaining useful life prediction based on similarity. *Comput. Sci. Eng.* **21**(1), 50–64. 10.1109/MCSE.2018.110145829 (2019).

[11] Shaheen, B. & Kocsis, N. I. Data-driven failure prediction and rul estimation of mechanical components using accumulative artificial neural networks. *Eng. Appl. Artif. Intell.* **119**, 105749. 10.1016/J.ENGAPPAI.2022.105749 (2023).

[12] Lee, J., Bagheri, B. & Kao, H.-A. Recent advances and trends of cyber-physical systems and big data analytics in industrial informatics. In *Int. Conference on Industrial Informatics (INDIN)* 1–6 . 10.13140/2.1.1464.1920 (2014).

[13] Jin, W., Liu, Z., Shi, Z., Jin, C. & Lee, J. Cps-enabled worry-free industrial applications. In *2017 Prognostics and System Health Management Conference, PHM-Harbin 2017 - Proceedings* 1–7. 10.1109/PHM.2017.8079208 (2017).

[14] Yan, J., Meng, Y., Lu, L. & Li, L. Industrial big data in an industry 4.0 environment: Challenges, schemes, and applications for predictive maintenance. *IEEE Access* **5**, 23484–23491. 10.1109/ACCESS.2017.2765544 (2017).

[15] Zhai, S., Kandemir, M. G. & Reinhart, G. Predictive maintenance integrated production scheduling by applying deep generative prognostics models: Approach, formulation and solution. *Prod. Eng. Res. Devel.* 1–24. 10.1007/s11740-021-01064-0 (2021).

[16] Baraldi, P., Di Maio, F., Al-Dahidi, S., Zio, E. & Mangili, F. Prediction of industrial equipment remaining useful life by fuzzy similarity and belief function theory. *Expert Syst. Appl.* **83**, 226–241 (2017).

[17] Xia, M., Zheng, X., Imran, M. & Shoaib, M. Data-driven prognosis method using hybrid deep recurrent neural network. *Appl. Soft Comput. J.* **93**, 106351. 10.1016/j.asoc.2020.106351 (2020).

[18] Li, J., Schaefer, D. & Milisavljevic-Syed, J. A decision-based framework for predictive maintenance technique selection in industry 4.0. *Procedia CIRP* **107**, 77–82. 10.1016/j.procir.2022.04.013 (2022).

[19] Mode, G. R., Calyam, P.& Hoque, K. A. False data injection attacks in internet of things and deep learning enabled predictive analytics. In *IEEE NOMS 2020 Conference* 11 (2019).

[20] Wu, D., Jennings, C., Terpenny, J., Kumara, S. & Gao, R. Cloud-based parallel machine learning for prognostics and health management: A tool wear prediction case study. *J. Manuf. Sci. Eng.* **140**, 10. 10.1115/1.4038002 (2017).

[21] Ayad, S., Terrissa, L.S. & Zerhouni, N. An IoT approach for a smart maintenance. In *2018 International Conference on Advanced Systems and Electric Technologies, IC_ASET 2018* 210–214. 10.1109/ASET.2018.8379861 (2018).

[22] Tao, F., Qi, Q., Liu, A. & Kusiak, A. Data-driven smart manufacturing. *J. Manuf. Syst.* **48**, 157–169. 10.1016/j.jmsy.2018.01.006 (2018).

[23] Kiangala, K. S. & Wang, Z. Initiating predictive maintenance for a conveyor motor in a bottling plant using industry 4.0 concepts. *Int. J. Adv. Manuf. Technol.* **97**(9–12), 3251–3271. 10.1007/s00170-018-2093-8 (2018).

[24] Chen, Y., Zhu, F. & Lee, J. Data quality evaluation and improvement for prognostic modeling using visual assessment based data partitioning method. *Comput. Ind.* **64**(3), 214–225. 10.1016/j.compind.2012.10.005 (2013).

[25] Cai, H., Feng, J., Li, W., Hsu, Y. M. & Lee, J. Similarity-based Particle Filter for Remaining Useful Life prediction with enhanced performance. *Appl. Soft Comput. J.* **94**, 106474. 10.1016/j.asoc.2020.106474 (2020).

[26] Lima, M. J. et al. HealthMon: An approach for monitoring machines degradation using time-series decomposition, clustering, and metaheuristics. *Comput. Ind. Eng.* **162**, 107709. 10.1016/j.cie.2021.107709 (2021).

[27] Zheng, S., Ristovski, K., Farahat, A. & Gupta, C. Long Short-Term Memory Network for Remaining Useful Life estimation. In *2017 IEEE International Conference on Prognostics and Health Management, ICPHM 2017* 88–95 (2017). 10.1109/ICPHM.2017.7998311

[28] Öztürk, E., Solak, A., Bäcker, D., Weiss, L. & Wegener, K. Analysis and relevance of service reports to extend predictive maintenance of large-scale plants. *Procedia CIRP* **107**, 1551–1558. 10.1016/j.procir.2022.05.190 (2022).

[29] Zonta, T. et al. A predictive maintenance model for optimizing production schedule using deep neural networks. *J. Manuf. Syst.* **62**, 452–462 (2022).

[30] Zhang, W., Yang, D. & Wang, H. Data-driven methods for predictive maintenance of industrial equipment: A survey. *IEEE Syst. J.* **13**(3), 2213–2227. 10.1109/JSYST.2019.2905565 (2019).

[31] Carvalho, T. P. et al. A systematic literature review of machine learning methods applied to predictive maintenance. *Comput. Ind. Eng.* **137**, 106024. 10.1016/j.cie.2019.106024 (2019).

[32] Bruneo, D. & De Vita, F. On the use of LSTM networks for predictive maintenance in smart industries. In *Proceedings - 2019 IEEE International Conference on Smart Computing, SMARTCOMP 2019* 241–248. 10.1109/SMARTCOMP.2019.00059 (2019).

[33] Dong, D., Li, X. Y. & Sun, F. Q. Life prediction of jet engines based on LSTM-recurrent neural networks. *2017 Prognostics and System Health Management Conference, PHM-Harbin 2017 - Proceedings* 1–6. 10.1109/PHM.2017.8079264 (2017).

[34] Heimes, F. O. Recurrent neural networks for remaining useful life estimation. In *2008 International Conference on Prognostics and Health Management, PHM 2008* 1–6. 10.1109/PHM.2008.4711422 (2008).

[35] Zonta, T. et al. Predictive maintenance in the Industry 4.0: A systematic literature review. *Comput. Ind. Eng.* **2020**, 106889. 10.1016/j.cie.2020.106889 (2020).

[36] Lim, P., Goh, C. K., Tan, K. C. & Dutta, P. Estimation of remaining useful life based on switching Kalman Filter neural network ensemble. In *PHM 2014 - Proceedings of the Annual Conference of the Prognostics and Health Management Society 2014* 2–9 (2014).

[37] Shi, J. et al. A dual attention LSTM lightweight model based on exponential smoothing for remaining useful life prediction. *Reliabil. Eng. Syst. Saf.* **243**, 109821. 10.1016/J.RESS.2023.109821 (2024).

[38] Lourari, A. W., Soualhi, A. & Benkedjouh, T. Advancing bearing fault diagnosis under variable working conditions: A ceemdan-sbs approach with vibro-electric signal integration. *Int. J. Adv. Manuf. Technol.* **132**(5), 2753–2772 (2024).

[39] Lourari, A. W., Yousfi, B. E. & Essaidi, A. B. Enhanced diagnosis of bearing and gear faults using Hilbert-Huang transform, singular value decomposition, and supervised learning methods. *Int. J. Adv. Manuf. Technol.* **139**(1), 983–999 (2025).

[40] Lourari, A. W., El Yousfi, B., Benkedjouh, T., Bouzar Essaidi, A. & Soualhi, A. Enhancing bearing and gear fault diagnosis: A vmd-pso approach with multisensory signal integration. *J. Vib. Control* **31**(19–20), 4098–4112 (2025).

[41] Lourari, A. W., Soualhi, A., Medjaher, K. & Benkedjouh, T. New health indicators for the monitoring of bearing failures under variable loads. *Struct. Health Monit.* **23**(5), 2922–2941 (2024).

[42] Lourari, A., Benkedjouh, T., El Yousfi, B. & Soualhi, A. An anfis-based framework for the prediction of bearing’s remaining useful life. *Int. J. Prognost. Health Manag.* **15**(1), 3791 (2024).

[43] Saleem, F., Umar, M. & Kim, J.-M. An optimized few-shot learning framework for fault diagnosis in milling machines. *Machines* **13**(11), 1010 (2025). 10.3390/s25185866 PMC12473162 41013110

[44] Umar, M., Saleem, F. & Kim, J.-M. Failure-consistent degradation state-space modeling of tool wear evolution and remaining useful life in machining systems. *Eng. Fail. Anal.* 110751 (2026).

[45] AI, M.A. Azure AI guide for predictive maintenance solutions. https://docs.microsoft.com/pt-br/azure/machine-learning/team-data-science-process/predictive-maintenance-playbook#solution-templates-for-predictive-maintenance (2020). Accessed 2020-09-08

[46] Aditya, B. M. Three Ways to Estimate Remaining Useful Life for Predictive Maintenance. Technical report, MathWorks. https://www.mathworks.com/company/newsletters/articles/three-ways-to-estimate-remaining-useful-life-for-predictive-maintenance.html (2018).

[47] Dudek, G. Pattern similarity-based methods for short-term load forecasting - part 1: Principles. *Appl. Soft Comput. J.* **37**, 277–287. 10.1016/j.asoc.2015.08.040 (2015).

[48] Jimenez-Cortadi, A., Irigoien, I., Boto, F., Sierra, B. & Rodriguez, G. Predictive maintenance on the machining process and machine tool. *Appl. Sci.* **10**, 224. 10.3390/app10010224 (2020).

[49] Scikit-learn: Metrics and scoring: quantifying the quality of predictions https://scikit-learn.org/stable/modules/model_evaluation.html#accuracy-score (2007). Accessed 2021-07-05

[50] Cui, Z., Chen, W. & Chen, Y. Multi-scale convolutional neural networks for time series classification. 10 arXiv:1603.06995 (2016).

